# Developmental Improvements and Persisting Difficulties in Children’s Metacognitive Monitoring and Control Skills: Cross‐Sectional and Longitudinal Perspectives

**DOI:** 10.1111/cdev.13486

**Published:** 2021-02-02

**Authors:** Natalie S. Bayard, Mariëtte H. van Loon, Martina Steiner, Claudia M. Roebers

**Affiliations:** ^1^ University of Bern

## Abstract

This study investigated age‐dependent improvements of monitoring and control in 7/8‐ and 9/10‐year‐old children. We addressed prospective (judgments of learning and restudy selections) and retrospective metacognitive skills (confidence judgments and withdrawal of answers). Children (*N* = 305) completed a paired‐associate learning task twice, with a 1‐year delay. Results revealed improvements in retrospective, but not in prospective monitoring and control. Furthermore, control remained suboptimal, seemingly a consequence of overoptimistic monitoring. Both age groups showed stronger monitoring‐based control at the second compared to the first assessment. The comparison with a cross‐sectional sample (*N* = 144) revealed that improvements in retrospective monitoring can be mainly attributed to naturally occurring development, whereas retrospective control seemed to improve due to increased task familiarity.

Imagine Sophie, 10 years old, studying for a Spanish vocabulary test. To achieve a good grade in the upcoming test, she should accurately evaluate which words she has learned sufficiently, and which ones she needs to restudy. During the test, Sophie has to check the spelling of each word and correct any possible errors. In literature, this ability to introspect, that is, to detect learning gaps and correct memory failures, is covered under the term of metacognition. One typically distinguishes between declarative metacognitive skills (knowledge about strategies, individual‐related and task‐related knowledge) and procedural metacognitive skills (Flavell, 1979). Procedural metacognitive skills entail monitoring (evaluation of ongoing cognitive activities: e.g., “How sure am I that I will remember the meaning of the word in the test?”; “How sure am I that the given answer is correct?”) and control (regulation of ongoing cognitive activities; e.g., selecting learning material for restudy; allocating study time depending on the learning material; withdrawing answers; Dunlosky & Metcalfe, 2009; Nelson & Narens, 1990). Depending on the timing within the learning process, different aspects of monitoring and control are distinguished. When acquiring new information, monitoring of the learning progress and controlling of the learning content are crucial. As for the example above, Sophie has to make judgments of learning (JOLs; prospective monitoring judgments) for each word, evaluating how sure she is that she will remember the meaning of the word in the test. She also has to decide which words she needs to restudy (prospective control) and ideally, she would base this decision on her JOLs (monitoring‐based control). During retrieval, that is, during Sophie’s test, she should retrospectively evaluate the correctness of every test response (give confidence judgments; CJs; retrospective monitoring) to identify and correct errors (withdrawal of previously given answers; retrospective control). Procedural metacognitive skills have consistently been documented to be important for many domains, as, for example, academic achievement (Schneider & Löffler, 2016) and self‐regulated learning (Dunlosky & Metcalfe, 2009), independent of age. Procedural metacognitive skills develop substantially during elementary school years (Schneider & Löffler, 2016). However, little is known about which aspects of monitoring and control (i.e., prospective vs. retrospective monitoring and control) develop earlier or faster, and which aspects persistently pose problems for children. To investigate intraindividual development of metacognitive skills and the stability of monitoring and control, longitudinal data are essential. To fill this gap in the literature, this study investigated different aspects of metacognitive development longitudinally; namely, by including two measurement points, two age groups, and two measures of monitoring as well as control. Since natural developments and practice effects (repeatedly testing the same individuals with similar types of tasks) are inherently confounded in longitudinal studies, it is impossible to unequivocally interpret changes across time as either true developmental improvements or mere practice effects or effects of task familiarity. To address this dilemma, we additionally conducted a cross‐sectional study in which participants were only tested once. In the present contribution, results of the longitudinal study will be reported as Study 1, and results of the comparison with the cross‐sectional study will be reported as Study 2.

## Development of Metacognitive Monitoring

Recent studies suggest that children as young as 3 years are—at least in principle—able to monitor their learning (Lipowski, Merriman, & Dunlosky, 2013; Lyons & Ghetti, 2013). In such studies, simple tasks and dichotomous monitoring scales are used to capture early signs of rudimentary monitoring skills. Monitoring is considered to be relatively accurate when participants give higher judgments for correct answers than for incorrect answers (referred to as “metacognitive monitoring resolution”, a classical measure of relative monitoring accuracy). Although even preschool and early elementary school children can basically distinguish between correct and incorrect recall in their monitoring judgments (Destan, Hembacher, Ghetti, & Roebers, 2014; Lipowski et al., 2013), children’s monitoring of incorrect recall remains far from perfect and undergoes a slow developmental progression during elementary school years (for recent reviews see Roebers, 2017; Schneider & Löffler, 2016). For one, monitoring of incorrect recall still tends to be error‐prone (judgments are overly optimistic; also referred to as “overconfidence”; Finn & Metcalfe, 2014). For another, monitoring the fine‐tuned differences between being “very unsure”, “unsure”, and “somewhat unsure” continues to pose problems until the end of elementary school years (Flavell, 2000).

Nelson and Narens’ (1990) theoretical framework of metacognition suggests that there is an overarching monitoring concept. In line with empirical evidence, however, a conceptual distinction between prospective (JOLs) and retrospective monitoring (CJs) may better explain patterns found for monitoring accuracy. The literature for both adults and children document higher accuracy for CJs compared to JOLs (Dougherty, Scheck, Nelson, & Narens, 2005; Maki, Shields, Wheeler, & Zacchili, 2005; Robey, Dougherty, & Buttaccio, 2017). Retrospective monitoring skills are present and substantial by the age of eight, but do further improve during elementary school (Roebers, 2002). Prospective monitoring, in contrast, generally tends to be less accurate and does not seem to develop at the same pace in the age range from 7 to 10 years (Metcalfe & Finn, 2013; Schneider, Visé, Lockl, & Nelson, 2000). The varying degrees of monitoring accuracy and the different patterns of developmental improvements in measures of monitoring underline the importance of distinguishing between prospective and retrospective monitoring judgments. The few existing cross‐sectional studies that directly compare prospective and retrospective monitoring suggest a higher consistency between JOLs and CJs in adults compared to children, and it seems that this consistency increases with age (Destan et al., 2014; Thiede & Dunlosky, 1994). The substantial correlations between JOLs and CJs point to shared processes, with retrievability of information certainly being one common process influencing both JOLs and CJs (Dougherty et al., 2005; Roebers, von der Linden, Schneider, & Howie, 2007). At the same time, these studies suggest that the consistency of JOL and CJ is far from perfect.

Together, although theoretically related, the evidence points to distinguishable monitoring aspects. The inclusion of prospective and retrospective monitoring judgments in two different age groups and at two different points in time allows exploring the commonalities and the differences as well as their developmental timetables. Differentiated and empirical insights into developmental progression of prospective and retrospective monitoring can advance the understanding of the underlying concept of metacognition. Furthermore, we can explore whether increasing monitoring skills merge to an overarching monitoring concept (Veenman & Spaans, 2005), or whether prospective and retrospective monitoring differentiate and follow different trajectories in the course of development. This is of practical importance both for teachers and teacher education, as detailed knowledge on different aspects of monitoring, a base for self‐regulated learning, can guide instructional methods in general and individual feedback.

## Development of Metacognitive Control and Monitoring‐Based Control

The ability to accurately monitor one’s own learning is fundamental because it affects the control of a learning process (Dunlosky & Metcalfe, 2009). Early signs of rudimentary control skills can be found in preschoolers. For example, children at this age are able to ask for help when they are uncertain about an answer (Coughlin, Hembacher, Lyons, & Ghetti, 2015), to select correct compared to incorrect responses more frequently for a final evaluation when receiving a reward (Hembacher & Ghetti, 2014), and to study difficult items longer than easy items (Destan et al., 2014). Further age‐related improvements in control skills have been observed until the end of elementary school years. These include study time allocation (Koriat, Ackerman, Lockl, & Schneider, 2009a; Lockl & Schneider, 2003), restudy selections (Metcalfe & Finn, 2013), and withdrawal or maintenance of previously given answers (Krebs & Roebers, 2010; Roebers, Krebs, & Roderer, 2014). Regarding the accuracy of these control skills, findings are somewhat inconsistent. While there is a recent study suggesting relatively well‐developed control skills in elementary school children (Lipowski, Ariel, Tauber, & Dunlosky, 2017), the majority of existing evidence suggests that suboptimal control skills continue to exist even in high school students (Dunlosky & Rawson, 2012; Wall, Thompson, Dunlosky, & Merriman, 2016). One of the reasons why metacognitive control is far from perfect may be inaccurate monitoring. For example, due to inaccurate monitoring, children may stop learning prematurely (if they are too optimistic) or restudy the wrong learning material. In fact, overconfident individuals tend to make more inadequate control decisions than less overconfident individuals (e.g., Destan & Roebers, 2015). Using monitoring judgments as basis for control is referred to as monitoring‐based control. However, accurate monitoring does not automatically lead to adequate control. Even when monitoring is relatively accurate, individuals may have difficulties transferring their monitoring into adequate control. This is typically observed when they base their control unsystematically on their monitoring (Roebers et al., 2014). Unsurprisingly, older elementary school children have been found to base their control more systematically on their monitoring judgments compared to younger children (Lockl & Schneider, 2003; Metcalfe & Finn, 2013; Roebers et al., 2014).

To date, the literature does not yet offer a comprehensive picture of developmental progression in monitoring‐based control. In some of the few existing studies, using simple perception or child‐appropriate paired‐associate tasks, relatively adequate monitoring‐based control has been documented in early elementary school children. Nevertheless, further improvements have been documented from elementary to secondary school (as indicted by a higher correlation between monitoring and control in older compared to younger children: Krebs & Roebers, 2010; Lipowski et al., 2017; Lockl & Schneider, 2003; Metcalfe & Finn, 2013). If monitoring‐based control is present early in development, then suboptimal control may be explained by inaccurate monitoring. Most previous studies included only one measure of monitoring‐based control (i.e., prospective or retrospective). To our knowledge, there is only one study examining both prospective and retrospective monitoring‐based control in preschoolers. They found that children showed more accurate retrospective compared to prospective monitoring‐based control (Destan et al., 2014). Given that prospective and retrospective monitoring develop differently and should therefore be distinguished, it is important to investigate if this also applies to prospective and retrospective monitoring‐based control. This study addressed monitoring‐based control both prospectively and retrospectively by linking two measures of monitoring to two different measures of control during learning and remembering.

## The Present Study

The focus of this study was to investigate developmental improvements in metacognitive monitoring and control as a function of age by including prospective and retrospective aspects. Existing evidence on developmental progression of monitoring (i.e., JOLs and CJs) and control relies mostly on cross‐sectional studies. To our knowledge, only two studies have examined metacognitive skills longitudinally, but addressed only one age group and one aspect of monitoring and control (Fandakova et al., 2017; Roebers & Spiess, 2017). The developmental perspective of this study allows examining different aspects of monitoring and control (i.e., prospective and retrospective) not only at a certain point in elementary school, but also over time and for two different age groups.

Based on previous findings, we hypothesized that fourth graders show more accurate monitoring (prospective and retrospective) than second graders. Furthermore, we aimed to test the hypothesis that retrospective monitoring is more accurate than prospective monitoring, independent of age (Dougherty et al., 2005; Roebers et al., 2014; Schneider et al., 2000). Regarding the correspondence between JOLs and CJs, we hypothesized a higher correspondence for the older compared to the younger age group, and for the second compared to the first measurement point, respectively (Veenman & Spaans, 2005).

Longitudinally, we hypothesized improvements in retrospective monitoring and in prospective and retrospective control in both age groups, but with nevertheless persisting suboptimal control (Metcalfe & Finn, 2013; Roebers & Spiess, 2017; Wall et al., 2016). Furthermore, we were interested in whether and how control relies on monitoring, referred to as monitoring‐based control (i.e., the relation between prospective monitoring and control, as well as the relation between retrospective monitoring and control). Based on the few existing cross‐sectional studies, we hypothesized fourth graders to show more accurate monitoring‐based control than second graders as well as improvements in monitoring‐based control over time, independent of age (Krebs & Roebers, 2010; Lipowski et al., 2017; Metcalfe & Finn, 2013).

To address these goals, a paired‐associates memory task was used to investigate monitoring and control in second and fourth graders; the age range for which the strongest improvements have been documented (Schneider & Löffler, 2016). The task was conducted twice, with a 1‐year break in between (Study 1). Because of the repeated presentation of the task (different items for each measurement point), possible improvements would have been difficult to interpret as either true developmental or mere practice effects. Therefore, we conducted Study 2 and compared the longitudinal sample from Study 1 with a cross‐sectional sample. Study 2 tested a sample of children that were within the same age range as the children in the sample of Study 1 at the second measurement point.

## Study 1

### Method

#### Overview

In Study 1, second and fourth graders had to solve a paired‐associates task twice, with a 1‐year break in between. At T_2,_ children were in third and fifth grade, respectively. To facilitate presentation of results, participants will be referred to as second and fourth graders, respectively, even after they had moved on to a higher grade at T_2._ At both measurement points, measures of recognition, monitoring (JOLs, CJs), and control (restudy selections, withdrawal of answers) were collected.

#### Participants

The total sample at T_1_ consisted of 305 children, including 140 second graders (50% girls; *M*_age_ = 7.6 years; *SD* = .5) and 165 fourth graders (47% girls; *M*_age_ = 9.6 years; *SD* = .5) from 12 different schools and 21 classes in the larger vicinity of a university town including rural and urban areas. The total sample at T_2_ consisted of 274 children, including 123 now third graders (52% girls) and 151 now fifth graders (48% girls). An attrition rate of 10% was caused by changes of residence and a few technical failures. Preliminary analysis for T_1_ revealed no significant differences between the children who dropped out and those who remained in terms of recall and metacognitive measures. Schools were recruited by contacting principals in the German speaking part of Switzerland. Children within a class were allowed to participate if parents signed an informed consent form prior to the study, and if they verbally agreed to participate before each testing. Family backgrounds were lower to upper middle class. Most of the participants were of European descent (93%). Overall, 66 children (22%) were non‐native speakers. However, they had sufficient languages skills in the local language to attend regular classes and to participate in the study. The research project named *Development of metacognitive skills in elementary school children aged between 8 and 11 years and the relation with metacognitive skills of teachers* from the Department of Psychology was approved by the Ethic Review Board of the Faculty of Humanities at the University of Bern, Switzerland, approval number: [2016‐08‐00004].

#### Materials and Procedure

**Figure 1:**
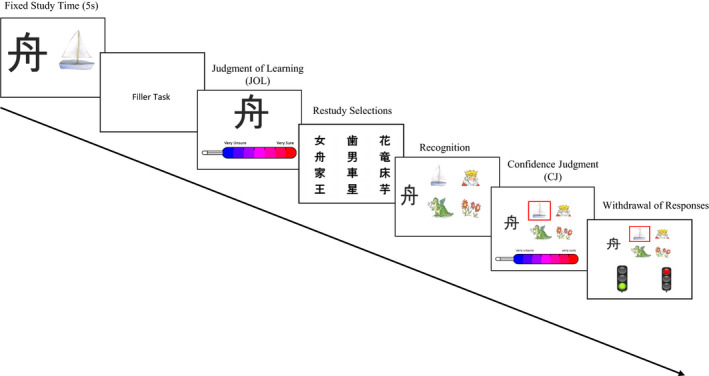
Schematic task procedure. [Colour figure can be viewed at wileyonlinelibrary.com]

Participants were tested in small groups of six to 11 children at their school. Testing lasted approximately 40 min. There were two experimenters present to ensure close assistance. The task was presented on a tablet computer. After receiving a general instruction from the experimenters, more detailed instructions were given orally via headphones and were additionally shown on the screen during the task. Before starting the task, children completed a practice trial to become familiar with the task and the tablets. The task was organized in five phases. Details of the experimental procedure are illustrated in Figure 1 [All materials can be obtained from the senior author upon request].

##### Study phase

During the study phase, 12 (for second grade) and 16 (for fourth grade) Japanese characters (Kanji) and their meanings (illustrated as colored line drawings) were randomly presented for 5 s. At T_2_, children learned a new set of Kanjis. No other modifications were made. The task had successfully been used in previous studies (Destan et al., 2014; Destan & Roebers, 2015; Roderer & Roebers, 2010). A large number of Kanji‐picture associations had been piloted before the project’s start. For each measurement point and for each age group, items were selected that varied in difficulty to ensure sufficient variability. Items with an item difficulty index (cf. Moosbrugger & Kelava, 2008) between 0.11 (difficult) and 0.78 (easy) were included in this study. Cronbach’s α for the 12 Kanjis for second graders was .40 and .62, for T_1_ and T_2_, respectively, and for the 16 Kanjis for fourth graders .46 and .71, for T_1_ and T_2_, respectively, indicating low to acceptable internal consistency. Differences in item difficulty and the relatively low number of items may have contributed to the lower level of internal consistency (Moosbrugger & Kelava, 2008), but are unavoidable in metacognitive research including children. After the study phase, a filler task was presented for 1 min to prevent rehearsal or other memory strategies. Because JOLs after a delay are more accurate than JOLs immediately after learning (the “delayed JOL effect”; Schneider et al., 2000), we measured JOLs after a short delay to best capture children’s emerging prospective monitoring skills.

##### Judgments of learning (prospective monitoring)

Children gave JOLs on a 7‐point Likert scale, illustrated with a colored thermometer (adapted from Koriat & Shitzer‐Reichert, 2002). The scale ranged from 1 (*very unsure*) to 7 (*very sure*). To provide a JOL, children responded to the following question: “*How sure are you that you will find the correct picture for this Kanji later on*?” At the start of the testing session, the thermometer was introduced with different example questions. Children learned to use the scales with ease. For assessing JOLs, one Kanji at a time was presented individually and without the corresponding picture. We chose this paradigm because JOLs are typically more accurate when the stimulus is presented without the response (Dunlosky & Nelson, 1992).

##### Restudy selections (prospective control)

All Kanjis from the study phase were presented together on one screen, and participants had the opportunity to select Kanjis for restudy by touching on the items. The instruction was as followed: *“If you could restudy one or more Kanjis to improve your later performance, which Kanjis would you select for restudy?”* Thereby, the position of the Kanjis on the screen was randomized. A simultaneous presentation of the items was chosen because prior studies found that the accuracy of restudy selections is higher when items were presented simultaneously compared to a sequential presentation (Thiede & Dunlosky, 1999; van Loon, de Bruin, van Gog, van Merriënboer, & Dunlosky, 2014). They could select any number of Kanjis. [There was no actual restudy phase after the selections, because this would have biased recognition and the relation between JOLs and performance. Children were not informed that there would be no actual restudy phase].

##### Recognition test and confidence judgments (retrospective monitoring)

Following the restudy phase (first control phase), children completed a recognition test. When taking the test, they had to choose the correct answer out of four alternatives. All alternatives were pictures that had appeared in the study phase and thus were familiar to the children. The recognition phase was conducted as a forced‐report phase. That is, even when unsure about the correct answers, children had to select an alternative. Once the child had selected an answer, a red frame surrounded it. Immediately afterward, the thermometer appeared below the alternatives for the second monitoring phase. Children were asked to provide CJs by answering the following question: “*How sure are you that you have chosen the correct picture?*”

##### Withdrawal or maintenance of answers (retrospective control)

At the end, participants were presented with their previously given answers, one at a time, and had the option to either maintain or withdraw their answer by pressing on a green or red traffic light. For this second control phase, children were told they could earn one point for accurate control (correct answer and green light), but that three points would be deducted for inaccurate control (incorrect answer and green light). The red light would neither give any points nor lead to negative consequences. This +1:−3 bonus‐to‐penalty scoring scheme is based on previous studies showing that elementary school children need a rather clear scoring scheme to trigger their control skills (e.g., Roebers, Schmid, & Roderer, 2009). These instructions were embedded in a cover story telling that the participating classes were competing against each other and that every class would get a feedback after finishing the assessment (a mock feedback with all classes ranking second to fifth within their age groups was sent out to every class after finalizing the measurement point).

Note that presentation times for the two monitoring phases, the recognition test and the two control phases, were self‐paced. Cronbach’s α for the different monitoring (JOLs and CJs) and control measures (restudy selections and withdrawal or maintenance of responses), calculated for each age group and each measurement point separately, ranged from .69 to .91, indicating acceptable to high internal consistency. At the end of the task, children were thanked for participation and were allowed to choose a small gift.

#### Dependent Variables

##### Recognition performance

Recognition performance was quantified by calculating the percentage of correct responses in the recognition test.

##### Metacognitive monitoring

For the analyses reported below, we calculated the difference between mean JOLs when responses in the recognition test were correct and mean JOLs when responses in the recognition test were incorrect, referred to as monitoring resolution in this study. The same was done with CJs. This is a commonly used measure of relative monitoring accuracy (Dunlosky & Thiede, 2013; Roebers & Spiess, 2017). Positive resolution values indicate that, on average, children gave higher monitoring judgments for correct than for incorrect responses.

##### Metacognitive control

For the analyses reported below, percentage of hits and misses for prospective and retrospective control were calculated. Hits in prospective control were defined as the number of incorrectly recognized items selected for restudy. Hits in retrospective control were the number of correct responses that were maintained. Misses in prospective control were defined as the number of incorrectly recognized items mistakenly not selected for restudy. Misses in retrospective control were the number correct responses mistakenly withdrawn (see Table 2). As hits and misses depend on a child’s recognition performance, hits and misses were calculated as individual percentages. For the analysis below, only hit rates for prospective and retrospective control were considered, given that miss rates are complement to hit rates (they add up to 100% and have the same variance).

##### Monitoring‐based control

For prospective and retrospective monitoring‐based control, Gamma correlations between monitoring judgments and the corresponding control indicator (i.e., within‐subject correlation between JOLs and restudy selections as well as between CJs and withdrawal or maintenance of responses, respectively) were calculated to investigate whether children systematically based their control on their monitoring judgments. Values can vary between −1 and +1, with higher values indicating more accurate monitoring‐based control (Nelson, 1984). A disadvantage of Gammas is that children without any variability in their monitoring judgments or control drop out of the analyses. Therefore, degrees of freedom can vary in the respective analyses (see below). Furthermore, children who very rarely varied their monitoring judgments can get extreme Gamma values (−1 or +1). Since those children would erroneously be considered as showing perfectly accurate monitoring‐based control, we excluded them from this set of the analyses. Based on these considerations, 13 children were excluded at T_1_ and 11 at T_2_, respectively, when considering prospective monitoring‐based control. For the corresponding analyses regarding retrospective monitoring‐based control, ten children were excluded at T_1_ and 13 at T_2_. [When including these few children with extreme Gamma values in the analyses, results remained very similar and conclusions remained the same.]

### Results

#### Recognition Performance

To provide insights into the underlying data structure of the metacognitive measures, we first report performance accuracy. Children in second grade recognized, on average, 45.1% (*SE* = 1.58) and 62.13% (*SE* = 1.86) of the items correctly at T_1_ and T_2_, respectively, and fourth graders recognized 54.8% (*SE* = 1.30) and 68.2% (*SE* = 1.61) correctly at T_1_ and T_2_, respectively. A mixed analysis of variance (ANOVA) with measurement point (T_1_ vs. T_2_) as within‐subject factor and age group (second vs. fourth) as between‐subjects factor revealed a significant improvement in performance over time, *F*(1, 272) = 123.52, *p* < .001, $\eta_{p}^{2}$ = .31. Furthermore, a main effect of age group with fourth graders performing better than second graders emerged, *F*(1, 272) = 19.51, *p* < .001, $\eta_{p}^{2}$ = .07. The interaction between measurement point and age group was not significant. Stability of performance was low to moderate with Pearson correlations between recognition at T_1_ and T_2_ being *r* = .19 (*p* = .039), for second graders, and *r* = .33 (*p* < .001), for fourth graders.

#### Prospective and Retrospective Monitoring

**Figure 2:**
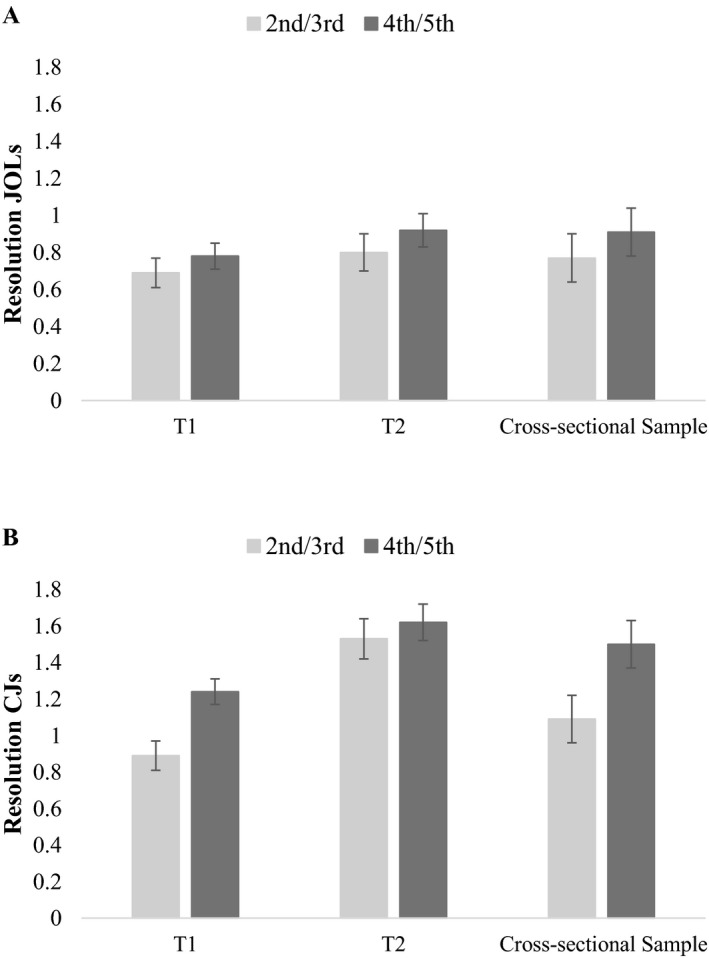
Mean resolution for judgments of learning (JOLs; A) and confidence judgments (CJs; B) as a function of measurement point and age group. Greater values indicate higher monitoring resolution between correct and incorrect responses. Error bars represent *SEM*.

As can be seen in Figure 2, monitoring resolution was not only more pronounced for retrospective than for prospective monitoring at T_1_, but resolution in retrospective monitoring also improved more strongly over time. The mixed ANOVA with judgment type (JOLs vs. CJs) and measurement point (T_1_ vs. T_2_) as within‐subject factors and age group (second vs. fourth grade) as between‐subjects factor confirmed our hypothesis that resolution was more pronounced for CJs (*M* = 1.32, *SE* = 0.05) than for JOLs (*M* = 0.80, *SE* = 0.04), *F*(1, 256) = 87.88, *p* < .001, $\eta_{p}^{2}$ = .26, and improved over time, *F*(1, 256) = 23.70, *p* = .001, $\eta_{p}^{2}$ = .09, (T_1_: *M* = 0.90, *SE* = 0.04; T_2_: *M* = 1.22, *SE* = 0.06). Furthermore, resolution values were higher for fourth than for second graders, *F*(1, 256) = 4.45, *p* = .036, $\eta_{p}^{2}$ = .02, and in line with our hypothesis, age differences were similar for JOLs and CJs. The significant interaction between judgment type and measurement point confirmed our hypothesis that the increase in resolution was stronger for CJs than for JOLs, *F*(1, 256) = 13.49, *p* < .001, $\eta_{p}^{2}$ = .05. All other interactions and the three‐way interaction were not significant, *F*s(1, 256) < 2.0, *p*s ≥ .180.

Exploratory Pearson correlations were calculated to address the consistency and stability of prospective and retrospective monitoring. Consistency across prospective and retrospective monitoring was calculated with Pearson correlations between JOL resolution and CJ resolution for T_1_ and T_2_ separately. Correlations were significant for both measurement points and both age groups (at *p* < .01), but only moderate (*r* = .26 and *r* = .24, for second graders at T_1_ and T_2_, respectively; *r* = .28 and *r* = .35 for fourth graders at T_1_ and T_2_, respectively). In contrast to our expectations, correlations did neither differ between age groups (Fisher’s *z* = −.19, *p* = .853), nor between measurement points (second graders: Fisher’s *z* = .17, *p* = .869; fourth graders: *z* = −.71, *p* = .476). Interestingly, stability over time, as measured with Pearson correlations of monitoring resolution within one type of monitoring across the two measurement points, was even lower. Neither prospective (second graders: *r* = −.02, *ns*; fourth grade: *r* = .16, *ns*) nor retrospective monitoring accuracy (second graders: *r* = .21, *p* < .03; fourth grade: *r* = .14, *ns*) were markedly stable.

#### Metacognitive Control

**Figure 3:**
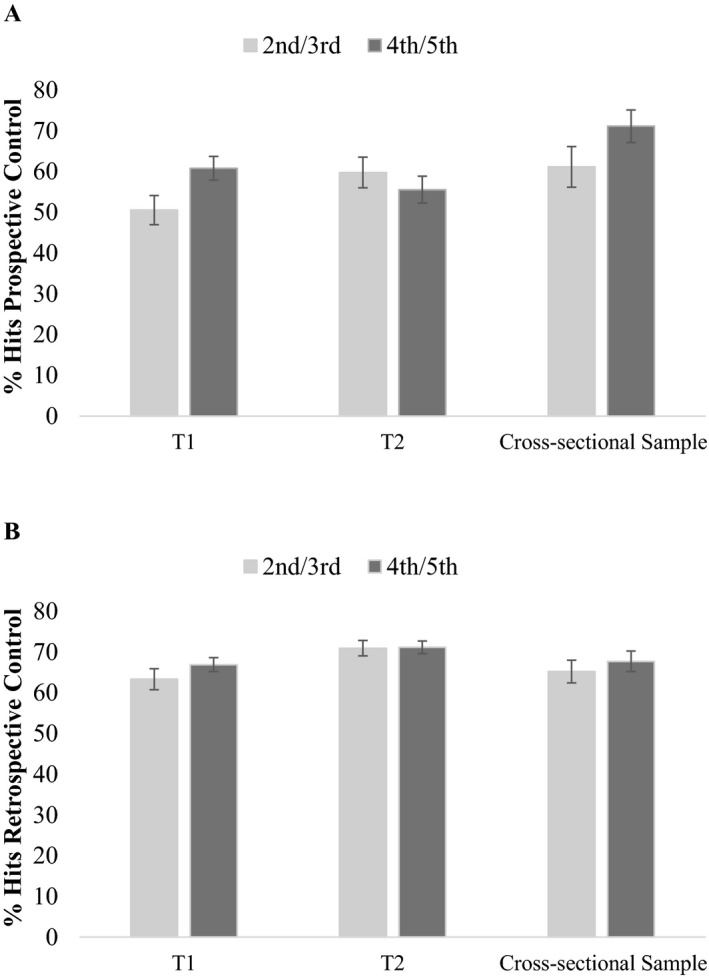
Mean percentages of hits for prospective (A) and retrospective (B) control as a function of measurement point and age group. Error bars represent *SEM*.

Children’s control was quantified by prospective selections of items for restudy and retrospective withdrawal or maintenance of previously given responses. As mentioned before, we investigated hits and misses for prospective and retrospective control decisions, but only hit rates will be considered here. Values of hit rates are represented in Figure 3. However, in the interest of completeness, all four control outcomes (i.e. hits, misses, correct rejections and false alarms) are shown in Table 1. Table 2 provides an overview over the different control outcomes. Furthermore, Table 3 shows the overall percentages of items selected for restudy and the percentages of items that were withdrawn. To investigate the percentages of hit rates, analyses were conducted separately for prospective and retrospective control, since instructions and methods were different for the two measures. Therefore, mixed analyses of variance (ANOVAs) with measurement point (T_1_ vs. T_2_) as within‐subject factors and age group (second vs. fourth grade) as between‐subjects factor were executed.

**Table 1:** Mean and Standard Errors for Hits, Misses, Correct Rejections, and False Alarms for Prospective and Retrospective Control in Study 1 and 2

	2nd/3rd grade		4th/5th grade	
	Prospective *M* (*SE*)	Retrospective *M* (*SE*)	Prospective *M* (*SE*)	Retrospective *M* (*SE*)
T_1_				
Hits %	50.56 (3.56)	63.30 (2.56)	60.80 (2.89)	66.87 (1.69)
Misses %	49.44 (3.56)	36.70 (2.56)	39.20 (2.89)	33.13 (1.69)
Correct rejections %	56.27 (3.11)	52.47 (2.86)	54.05 (2.46)	66.53 (2.41)
False alarms %	43.73 (3.11)	47.53 (2.86)	45.95 (2.46)	33.47 (2.41)
T_2_				
Hits %	59.77 (3.76)	70.91 (1.87)	55.56 (3.32)	71.15 (1.56)
Misses %	40.23 (3.76)	29.09 (1.87)	44.44 (3.32)	28.85 (1.56)
Correct rejections %	57.04 (3.20)	70.44 (2.93)	60.94 (2.50)	78.15 (2.22)
False alarms %	42.96 (3.20)	29.56 (2.93)	39.06 (2.50)	21.85 (2.22)
Cross‐sectional sample				
Hits %	61.16 (5.00)	65.15 (2.79)	71.14 (4.00)	67.68 (2.50)
Misses %	38.84 (5.00)	34.85 (2.79)	28.86 (3.99)	32.32 (2.50)
Correct rejections %	56.20 (4.61)	64.14 (4.02)	43.73 (3.67)	76.07 (2.98)
False alarms %	43.80 (4.61)	35.86 (4.02)	56.27 (3.67)	23.93 (2.98)

Note

Hits, misses, correct rejections, and false alarms depend on the performance and are therefore calculated with respect to the performance of each child (hits and misses; correct rejections and false alarms add up to 100% and have the same variance).

**Table 2:** Definition of Hits, Misses, Correct Rejections and False Alarms for Prospective and Retrospective Control

	Prospective control	
	Item selected for restudy	Item not selected for restudy
Correct response	False alarm	Correct rejection
Incorrect response	Hit	Miss

	Retrospective control	
	Item withdrew	Item maintained
Correct response	Miss	Hit
Incorrect response	Correct rejection	False alarm

**Table 3:** Mean Percentage and Standard Errors of Mean of Items Selected for Restudy (Prospective Control) and of Withdrawn Items (Retrospective Control) at T_1_ and T_2_

	T_1_ *M* (*SE*)	T_2_ *M* (*SE*)	Cross‐sectional sample *M* (*SE*)
2nd/3rd grade			
Items selected for restudy	47.36 (3.11)	48.71 (3.17)	50.36 (4.44)
Items withdrawn	48.04 (2.31)	42.81 (2.02)	46.55 (2.76)
4th/5th grade			
Items selected for restudy	47.48 (2.33)	44.16 (2.53)	60.47 (3.50)
Items withdrawn	47.68 (1.75)	41.47 (1.68)	45.44 (2.44)

Note

The maximum number of items to select for restudy or to withdraw was 12 for 2nd/3rd graders and 16 for 4th/5th graders.

Regarding prospective control, the main effects of measurement point, *F*(1, 257) = 0.12, *p* = .730, and age group, *F*(1, 257) = 0.32, *p* = .570, were not significant. Surprisingly and in contrast to our hypothesis, a significant interaction between measurement point and age group emerged, *F*(1, 257) = 5.80, *p* = .017, $\eta_{p}^{2}$ = .02, indicating higher hit rates for second graders at T_2_ than T_1_, whereas fourth graders had fewer hit rates at T_2_ than T_1_.

When addressing retrospective control, our hypothesis was confirmed. Analysis of variance revealed a significant main effect of measurement point, *F*(1, 271) = 13.73, *p* < .001, $\eta_{p}^{2}$ = .05, showing higher hit rates at T_2_ (*M* = 71.03, *SE* = 1.21) than T_1_ (*M* = 65.01, *SE* = 1.49). Neither the main effect of age group, *F*(1, 271) = 0.71, *p* = .400, nor the interaction between measurement point and age group, *F*(1, 271) = 0.95, *p* = .330, were significant.

Stability, indexed with exploratory correlations of hit rates within one type of control over time, was moderate for prospective control (*r* = .39 and *r* = .36, for second and fourth graders, respectively, *p* < .001), as well as for retrospective control (*r* = .27 and *r* = .32, for second and fourth graders, respectively, *p* < .001).

#### Monitoring‐Based Control

To test our hypotheses regarding monitoring‐based control, within‐subject correlations (Gammas) between the monitoring judgments and the corresponding control were calculated for each participant. All mean Gamma correlations for both measurement points and both age groups were significantly different from zero, *t*s ≥ 11.10, *p*s < .001, indicating that control was—at least to some extent—systematically based on monitoring judgments.

**Figure 4:**
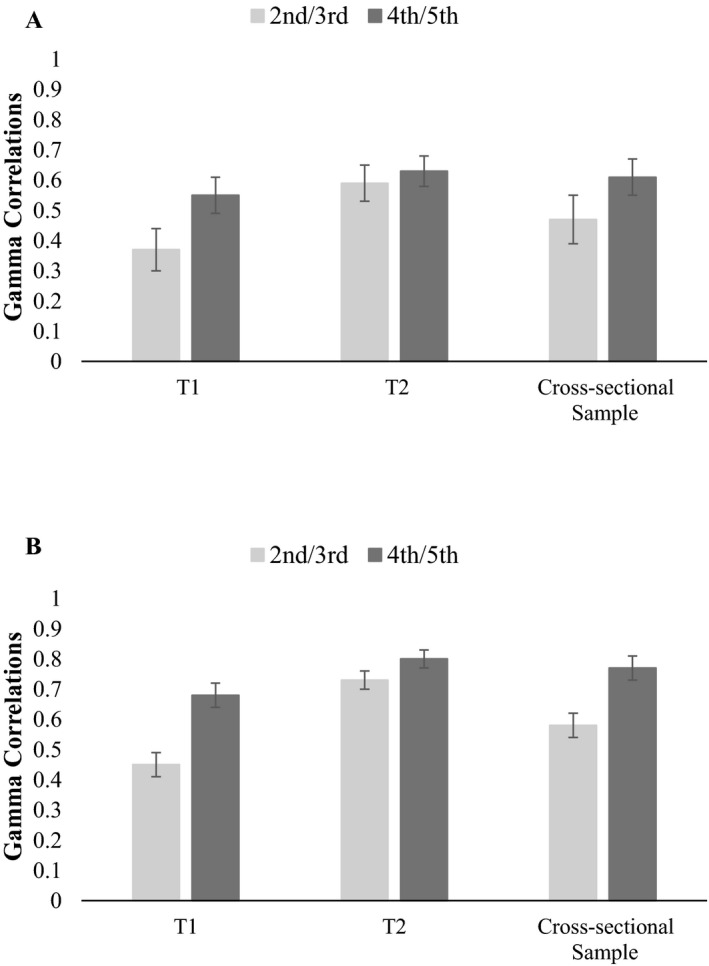
Gamma correlations for judgments of learning and prospective control (A) as well as for confidence judgments and retrospective control (B) as a function of measurement point and age group. Error bars represent *SEM*.

Addressing Gammas between JOLs and prospective control, analyses revealed a significant main effect of measurement point, *F*(1, 156) = 8.76, *p* = .004, $\eta_{p}^{2}$ = .05, and age group *F*(1, 156) = 6.54, *p* = .012, $\eta_{p}^{2}$ = .04. Figure 4A shows, in line with our hypotheses, that Gammas were higher at T_2_ (*M* = 0.60, *SE* = 0.04) than at T_1_ (*M* = 0.45, *SE* = 0.04), and higher in fourth graders (*M* = 0.60, *SE* = 0.04) than in second graders (*M* = 0.44, *SE* = 0.05). The interaction between measurement point and age group was not significant, *F*(1, 156) = 1.70, *p* = .195.

Gammas between CJs and retrospective control are presented in Figure 4B. As hypothesized, significant main effects of age group, *F*(1, 217) = 11.83, *p* < .001, $\eta_{p}^{2}$ = .05, and measurement point were found, *F*(1, 217) = 54.18, *p* < .001, $\eta_{p}^{2}$ = .20, revealing that Gamma correlations were higher in fourth graders (*M* = 0.74; *SE* = 0.03) than in second graders (*M* = 0.60, *SE* = 0.03), and higher at T_2_ (*M* = 0.76, *SE* = 0.02) than at T_1_ (*M* = 0.57, *SE* = 0.03). Interestingly, and in contrast to prospective monitoring‐based control, a significant interaction between measurement point and age group emerged, *F*(1, 217) = 9.78, *p* = .002, $\eta_{p}^{2}$ = .04, indicating that the increase in Gammas over time was stronger for second than for fourth graders.

#### Persisting Metacognitive Difficulties

Finally, we exploratively investigated the relation between recognition performance, monitoring judgments, and control decisions to shed light on reasons why control failures occurred. In general, it is expected that individuals give lower monitoring judgments (i.e., JOLs and CJs) to incorrect responses and will then decide to restudy that item or withdraw that response more often compared to correct responses. Based on this expectation, we explored whether children were able to accurately monitor responses that (a) they had the intention to restudy or withdrew as well as those that (b) they chose not to restudy or decided to maintain. For these analyses, mixed ANOVAs were conducted separately for prospective and retrospective control and for the second measurement point only.

**Figure 5:**
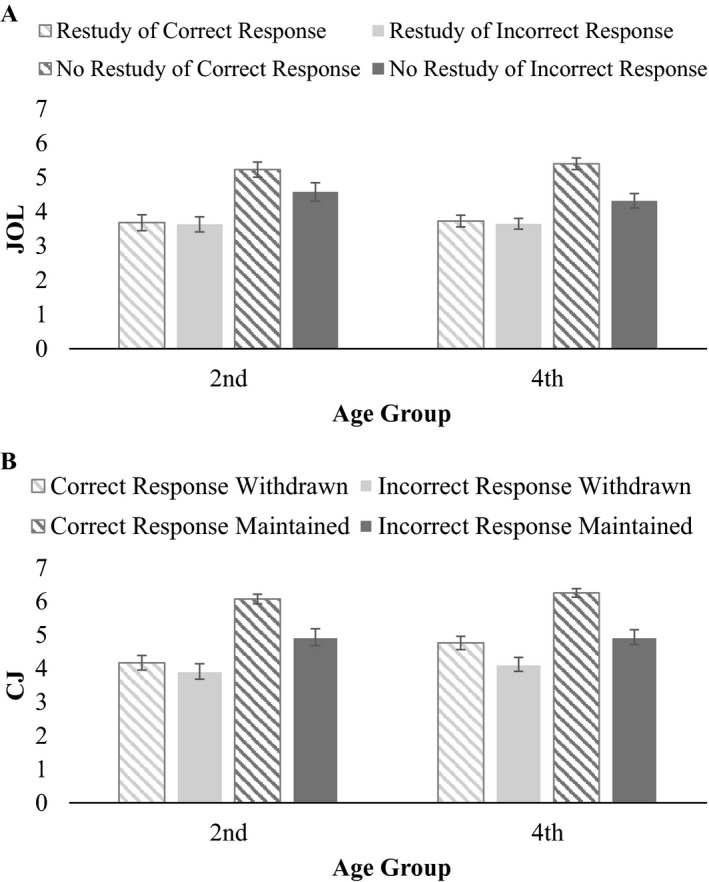
Mean judgments of learning (JOL) for correct and incorrect responses (A) and mean confidence judgment (CJ) for correct and incorrect responses (B) as a function of age group and control decision (A: restudy vs. no restudy; B: maintained vs. withdrawn) for T_2._ *SE*s are presented by the error bars.

Regarding prospective control, we performed an exploratory mixed ANOVA with correctness (correctly vs. incorrectly recognized item) and control decision (item selected for restudy vs. not selected for restudy) as within‐subject factors and age group (second vs. fourth graders) as between‐subjects factor. JOLs (1–7) served as dependent variable. Results are shown in Figure 5A and revealed a significant main effect of correctness, *F*(1, 92) = 15.01, *p* < .001, $\eta_{p}^{2}$ = .14, indicating higher JOLs for correctly recognized items (*M* = 4.51, *SE* = 0.10) compared to incorrectly recognized items (*M* = 4.05, *SE* = 0.13). Furthermore, items that children selected for restudy had lower JOLs (*M* = 3.67, *SE* = 0.13) than items not selected for restudy (*M* = 4.89, *SE* = 0.12), *F*(1, 92) = 88.03, *p* < .001, $\eta_{p}^{2}$ = .49. The main effect of age group was not significant, *F*(1, 92) = 0.00, *p* = .972. Finally, a significant interaction between correctness and control decision was found, *F*(1, 92) = 15.50, *p* < .001, $\eta_{p}^{2}$ = .14. While for the nonselected items, children discriminated between correct and incorrect responses in their JOLs (i.e., lower JOLs for incorrect than for correct responses), no difference was found in JOLs between correct and incorrect responses for the selected items. In other words, JOLs were more strongly associated with restudy selections than with recognition accuracy, demonstrating that children strongly relied on their monitoring, above and beyond recognition performance. All other interactions were not significant, *F*s(1, 92) < 2.0, *p*s ≥ .314.

Concerning retrospective control, an exploratory mixed ANOVA was calculated with correctness (correct vs. incorrect responses) and control decision (item maintained vs. withdrawn) as within‐subject factors and age group (second vs. fourth graders) as between‐subjects factor. CJs (1–7) served as dependent variable. Results are shown in Figure 5B. Significant main effects of correctness, *F*(1, 106) = 53.58, *p* < .001, $\eta_{p}^{2}$ = .34, and control decision, *F*(1, 106) = 115.06, *p* < .001, $\eta_{p}^{2}$ = .52, emerged, indicating higher CJs for correct responses (*M* = 5.31, *SE* = 0.10) than for incorrect responses (*M* = 4.48, *SE* = 0.13), and higher CJs for maintained (*M* = 5.55, *SE* = 0.12) than for withdrawn items (*M* = 4.24, *SE* = 0.13). The main effect of age group was not significant, *F*(1, 106) = 1.34, *p* = .249. Again, a significant interaction between correctness and control decision was found, *F*(1, 106) = 15.90, *p* < .001, $\eta_{p}^{2}$ = .13, showing that children’s monitoring was relatively adequate for the maintained responses, whereas monitoring resolution was poor for the withdrawn items, irrespective of correctness of recognition. All other interactions were nonsignificant, *F*s(1, 106) < 2.0, *p*s ≥ .208.

### Discussion

Study 1 confirmed the existing evidence regarding monitoring: there were (a) substantial age differences in monitoring and control, (b) significant improvements in monitoring (Krebs & Roebers, 2010; Roebers, 2002; Schneider & Löffler, 2016), and (c) indications of overall more accurate retrospective monitoring than prospective monitoring (Dougherty et al., 2005; Maki et al., 2005; Roebers et al., 2007). This study provided new and detailed insights into elementary school children’s metacognitive improvements that go beyond previous findings. As expected, both second and fourth graders were found to improve their retrospective monitoring over the course of 1 year, while their prospective monitoring did not improve, at least not on group level. Regarding control, retrospective control became more accurate over time in both age groups, whereas prospective control improved only in second graders. This result is surprising, given that we expected improved control for both measures and independent of age. We will discuss this further as part of the general discussion. Another important finding was that monitoring‐based control was present in both second and fourth graders, which is in line with previous findings (Krebs & Roebers, 2010; Lipowski et al., 2017; Metcalfe & Finn, 2013). Our study provided first evidence for developmental improvements in prospective monitoring‐based control independent of age, and stronger improvement in retrospective monitoring‐based control in the younger compared to the older age group.

Interpreting developmental improvements in metacognitive skills leads straight into a dilemma. In fact, it lies in the nature of development that the emergence of more sophisticated functioning is inextricably confounded with practice effects due to the repeated presentation of the task. In other words, it remains yet an open question whether children’s metacognitive skills improved due to natural development or rather because they were familiar with the task at the second measurement point due to previous practice. To address this question and to allow a more conclusive interpretation of the longitudinal effects documented above, we added a cross‐sectional study, investigating the same metacognitive skills in children of this age. We expected that although there would be practice effects, that is, better performance in the longitudinal compared to the cross‐sectional sample, most of the observed improvements would be attributable to true developmental improvements rather than to mere practice. In other words, we hypothesized that children from the longitudinal sample show higher recognition performance compared to the cross‐sectional sample, and that the two samples do not differ in the accuracy of monitoring and control decisions.

## Study 2

### Overview

In Study 2, a sample of children (third and fifth graders) with a comparable age to children from the longitudinal sample at T_2_, was investigated. For practical reasons, the Study 1 sample will be referred to as the longitudinal sample in the following section and the Study 2 sample as cross‐sectional sample.

### Participants and Method

The cross‐sectional sample consisted of 70 third graders (53% girls; *M*_age_ = 8.5; *SD* = .5) and 74 fifth graders (46% girls; *M*_age_ = 10.6; *SD* = .5) from five schools and nine classes. Family backgrounds were comparable to the longitudinal sample (lower to upper middle class). The longitudinal sample was the same as in Study 1 at T_2_. It consisted of 123 third graders (52% girls; *M*_age_ = 8.6; *SD* = .53) and 151 fifth graders (48% girls; *M*_age_ = 10.7; *SD* = .5). The cross‐sectional data were collected 1 year after T_2_ data collection for the longitudinal study. The method and the presented items (Kanjis) were identical to T_2_ in Study 1. Readers are reminded that sample sizes can vary in the analyses due to an inability to compute within‐person Gamma correlations when no variability was observed (e.g., when children gave only the highest confidence ratings throughout). As in Study 1, children who very rarely varied their monitoring judgments were excluded from the analyses for monitoring‐based control. For the cross‐sectional sample, nine children were excluded for prospective monitoring‐based control and seven children for retrospective monitoring‐based control.

### Results

#### Recognition Performance

In the cross‐sectional sample, third graders correctly recognized on average 55.36% (*SE* = 2.49) and fifth graders 65.37% (*SE* = 2.30) of the items. Possible differences in recognition performance between the cross‐sectional and the longitudinal sample were examined running a two‐way ANOVA with age group (third vs. fifth grade) and sample (cross‐sectional vs. longitudinal sample) as factors and percentage of correct recognition as dependent variable. Analysis showed that older children outperformed younger children, *F*(1, 414) = 14.93, *p* < .001, $\eta_{p}^{2}$ = .04, and the longitudinal sample outperformed the cross‐sectional sample, *F*(1, 414) = 5.32, *p* < .001, $\eta_{p}^{2}$ = .01, giving hints to task familiarity effects rather than true developmental improvements when considering memory performance. The finding is in line with our hypothesis. The interaction was not significant, *F*(1, 414) = 0.89, *p* = .346.

#### Prospective and Retrospective Monitoring

Monitoring resolution for prospective (JOLs) and retrospective (CJs) monitoring judgments are depicted in Figure 2 and were entered into a mixed ANOVA with judgment type (JOLs vs. CJs) as within‐subject factor and age group (third vs. fifth grade) as well as sample (cross‐sectional vs. longitudinal sample) as between‐subjects factors. As in Study 1, monitoring resolution was more pronounced for CJs (*M* = 1.44; *SE* = 0.06) than for JOLs (*M* = 0.85; *SE* = 0.06), *F*(1, 390) = 74.40, *p* < .001, $\eta_{p}^{2}$ = .16. Furthermore, monitoring resolution was stronger in fifth graders (*M* = 1.24; *SE* = 0.07) compared to third graders (*M* = 1.05; *SE* = 0.07), *F*(1, 390) = 4.01, *p* = .046, $\eta_{p}^{2}$ = .01. As hypothesized, the main effect of sample and all interactions were not significant, *F*s(1, 390) ≤ 3.69, *p*s ≥ .056. Since there was no difference in prospective and retrospective monitoring resolution between the longitudinal and cross‐sectional sample at T_2_, the observed improvements in monitoring in Study 1 suggest true development rather than simple practice effects.

#### Metacognitive Control

For the evaluation of metacognitive control, hit rates for prospective and retrospective control were examined separately. Values are shown in Figure 3 and Table 1. Two‐way ANOVAs with age group (third vs. fifth grade) and sample (cross‐sectional vs. longitudinal sample) as between‐subjects factors and percentage of hit rates as dependent variable were conducted. Against our hypothesis, the main effect of sample for prospective control was significant, *F*(1, 391) = 4.19, *p* = .041, $\eta_{p}^{2}$ = .01. Hit rates were higher for the cross‐sectional sample (*M* = 66.15; *SE* = 3.35) than the longitudinal sample (*M* = 57.67; *SE* = 2.44). The main effect of age group and the interaction were not significant, *F*s(1, 391) ≤ 2.93, *p*s ≥ .088. In contrast, analyses of retrospective control showed that the longitudinal sample (*M* = 71.03; *SE* = 1.26) had higher hit rates compared to the cross‐sectional sample (*M* = 66.41; *SE* = 1.73), *F*(1, 413) = 4.64, *p* = .032, $\eta_{p}^{2}$ = .01. This result indicates that the observed improvements in retrospective control in Study 1 are mostly due to practice effects rather than to actual development. The main effect of age group and the interaction were not significant, *F*s(1, 413) ≤ 0.42, *p*s ≥ .518.

#### Monitoring‐Based Control

Monitoring‐based control, presented in Figure 4, was investigated by calculating Gamma correlations between the monitoring judgment and the corresponding control decision, as was longitudinally addressed in Study 1. All Gammas were significantly different form zero, *t*s ≥ 5.44, *p*s ≤ .001, suggesting that children systematically based their control decisions on their monitoring judgments, similar to Study 1. Conforming our hypothesis, the conducted two‐way ANOVA with age group (third vs. fifth grade) and sample (cross‐sectional vs. longitudinal sample) as factors and Gamma correlations between JOLs and prospective control as dependent variable revealed that neither the main effects nor the interaction were significant, *F*s(1, 279) ≤ 2.03, *p*s ≥ .154. Thus, the improvements in prospective monitoring‐based control found in Study 1 indicate true developmental improvements rather than practice effects. In the corresponding analyses for retrospective monitoring‐based control, Gammas were higher in fifth graders (*M* = 0.77; *SE* = 0.03) than in third graders (*M* = 0.64; *SE* = 0.03), *F*(1, 370) = 12.53, *p* ≤ .001, $\eta_{p}^{2}$ = .03, and higher in the longitudinal sample (*M* = 0.75; *SE* = 0.02) compared to the cross‐sectional sample (*M* = 0.67; *SE* = 0.03), *F*(1, 370) = 5.63, *p* = .018, $\eta_{p}^{2}$ = .02, which is against our hypothesis. Furthermore, the interaction between age group and sample was significant, *F*(1, 370) = 4.05, *p* = .045, $\eta_{p}^{2}$ = .01, indicating that age differences were greater in the cross‐sectional than in the longitudinal sample. The difference in retrospective monitoring‐based control between longitudinal and cross‐sectional sample points out that the observed improvement in Study 1 is rather due to practice than development.

## General Discussion

The goal of this study was to investigate developmental progression of monitoring and control skills in elementary school children with the specific aim to compare the accuracy and the development of prospective (JOLs) and retrospective monitoring (CJs) and control (restudy selections and response withdrawal). The longitudinal sample’s memory performance and metacognitive skills were additionally compared to a cross‐sectional sample to estimate the effects of practice and task familiarity. As the literature consistently documents suboptimal control in elementary school years (Schneider & Löffler, 2016), we also aimed to shed light on children’s persisting metacognitive difficulties by exploring the contribution of monitoring inaccuracy for metacognitive control failures.

### Metacognitive Monitoring

A major strength of this study is the combination of different measures of monitoring within one task. Comparing prospective and retrospective monitoring provides insights into the internal structure of monitoring, an important aspect for advancing the theoretical notions of the underlying construct. For applied contexts, it allows gaining better understanding of the specific difficulties that children face when monitoring their performance. Namely, by uncovering different developmental timetables for various indicators of metacognition. The substantial correlation between prospective and retrospective monitoring found in previous studies suggests an underlying common factor (Destan et al., 2014; Thiede & Dunlosky, 1994). In this study, the instructions and scales on which children provided the judgments were identical and this, together with the fact that the two measures are theoretically related (Destan et al., 2014; Dougherty et al., 2005; Roebers et al., 2007), allowed a direct comparison. As reported in studies with adults (Dougherty et al., 2005; Maki et al., 2005; Robey et al., 2017) and confirming our hypothesis, the comparisons revealed an overall higher accuracy for retrospective monitoring than for prospective monitoring. Moreover, we found that older children were more accurate in both prospective and retrospective monitoring than younger children, which was in line with our hypotheses. In general, however, accuracy of prospective monitoring was rather low, suggesting that children’s ability to predict their performance of individual items follows a longer developmental trajectory.

The inclusion of two monitoring measures also allowed addressing consistency of monitoring skills. On the one hand, the correspondence between prospective and retrospective monitoring accuracy was low to moderate. Together with the finding that prospective and retrospective monitoring developed differently, this suggests a heterogeneous rather than a homogeneous, overarching monitoring construct. On the other hand, however, the stability of prospective and retrospective monitoring accuracy over time was low as well. A reason for the low stability of monitoring accuracy may be the low reliability of recognition performance as monitoring heavily relies on recognition. Reliability of recognition performance and thus the stability of monitoring accuracy could possibly be improved by including a larger number of items. This would make the perceptual input of the items more similar to avoid fluctuating, item‐specific strategies, and reduce the probability of guessing in the recognition test. Hence, more studies are needed to theoretically and empirically clarify the very nature of the monitoring concept.

Furthermore, only retrospective monitoring improved over time. One the one hand, there were no significant differences in monitoring accuracy between the longitudinal and the cross‐sectional sample, suggesting that the observed improvements in retrospective monitoring reflect developmental changes rather than practice effects. On the other hand, however, this interpretation must be treated with caution as there were rather large numerical differences between the two samples and we found evidence for practice effects in retrospective control as well as in retrospective monitoring‐based control. Thus, the observed improvements in retrospective monitoring are probably the result of both naturally occurring development and practice.

Together, the findings uncovered different developmental timetables for prospective and retrospective monitoring. This is the first study providing direct evidence for this claim. Importantly, different trajectories suggest different demands and monitoring processes to be involved. To provide a JOL implies to actively retrieve the corresponding meaning from memory (as the meaning was not shown), whereas giving CJs “only” demands the recognition of the correct answer. Consequently, some children might have indicated being very unsure when providing JOLs, because they could not recall the corresponding picture at the moment, but when having the possibility to recognize the correct picture out of a choice, they may have used other strategies like guessing or selecting the best answer. This could have led to more accurate retrospective than prospective monitoring.

Detailed knowledge concerning the development in prospective and retrospective monitoring is not only theoretically crucial but also practically important. For one, our findings are relevant for teachers and teacher education, reaching from informing teachers about naturally occurring developments and their timetables, to issues of instructional methods. That is, the present results can inform practitioners when to instruct, foster and give feedback to students concerning what aspect of their learning. For another, it can directly help teachers to understand difficulties and to specifically promote those aspects of monitoring that typically pose problems, e.g. building on retrospective, earlier developing and trainable skills to help children improve their prospective monitoring. In this context, it may be beneficial if teachers create tasks and situations within which children can make multiple and repeated experiences with monitoring, evaluate their monitoring to detect monitoring failures, and receive feedback from teachers about their monitoring accuracy. This study suggests that it may be best to start with fostering retrospective monitoring skills, because prospective monitoring still seems to be difficult for children in elementary school and there was no evidence that practice alone led to improvements. Thus, teachers should foster monitoring as part of a test, for example, by finishing the test with an additional self‐monitoring phase for all children. Taking it one further step, and because monitoring is also inherent in many everyday life tasks, teachers may want to consider raising parents’ awareness of monitoring and its importance (its nature, when and where it can take place, what are the cornerstones of accurate monitoring and the like). This way, parents might be empowered to address monitoring in activities of their daily life (when playing memory games, when planning a trip), increasing the number and the variety of children’s metacognitive experiences. Coming back to the introductory example of Sophie, it would be helpful for Sophie if one of her parents (or any other person at home) would assist in taking a mock test, articulate when she responds promptly, when she hesitates, which words she got right and which answers were wrong. At school, ideally, Sophie and her classmates should have the possibility to evaluate their responses after taking the Spanish vocabulary test and to get feedback about their overall performance as well as about the accuracy of their self‐evaluations.

The heterogeneity and fluctuations of monitoring skills found in the present approach seem the rule rather than the exception (Flavell, 2000). The cue utilization framework (Koriat, 1997) provides one explanation for why individuals’ monitoring skills vary among tasks, kinds of judgments, and kind of memory probe. Cue utilization refers to the assumption that individuals of any age base their judgments on different cues (e.g., familiarity, invested learning effort, difficulty; Koriat, 1997). In this study, children may have used different cues for prospective (e.g., item difficulty) and retrospective monitoring (e.g., retrieval fluency in recognition test). These cues may also occur with different frequencies in children’s everyday life. Independent of frequency, some cues are easier to detect for children than others, (Koriat, Ackerman, Lockl, & Schneider, 2009b) leading to different developments in prospective and retrospective monitoring. However, given that we did not quantify cue utilization for the different indicators of metacognition, this interpretation remains somewhat speculative (but see Roebers, Mayer, Steiner, Bayard, & van Loon, 2019).

### Metacognitive Control

With respect to metacognitive control, we expected improvements for both age groups and both measures. Unexpectedly, prospective control improved in second graders only, whereas fourth graders had even lower hit rates at T_2_ compared to T_1_. Readers are reminded that the analyses included only percentages of hits (incorrectly recognized item selected for restudy). Importantly, however, an adequate control decision is also made when children do not select an item for restudy that they later recognized correctly (correct rejections). In fact, when taking hits and correct rejections together, overall accuracy of control was comparable at T_1_ and T_2_ for fourth graders, indicating that older children neither improved nor became worse over time. One explanation for this pattern could lie in children’s motivation. We did not inform participants that there was no restudy phase. However, it is likely that at T_2_, participants remembered these details. Therefore, children might have been less motivated to reflect at later compared to earlier task completions about restudying, leading to fewer hit rates, at least in the older group. This explanation is supported by Table 3 revealing that fourth graders overall selected fewer items for restudy at T_2_ compared to T_1_, especially in contrast to second graders. Another reason may be the bonus‐penalty system that was applied for retrospective control only, making retrospective control decision more relevant and motivating. Furthermore, prospective control implied—at least as perceived by our participants—that they needed to invest more study time, a prospect that both second and fourth graders might not have found attractive. These motivational interpretations are supported by the fact that prospective control was more adequate in the cross‐sectional sample compared to the longitudinal sample. As the study’s goal was not to investigate motivational effects on control, we applied two somewhat different control instructions that may now, for direct comparisons, be considered as a limitation of this study. However, to address stability over time, age differences, and developmental progression, the present approach proved to be well suited. Future research should systematically investigate motivational effects on children’s restudy selections.

In contrast to prospective control, we found the expected improvements over time in retrospective control for both age groups. The comparison with the cross‐sectional sample showed that the observed improvements were more likely due to practice effects than to genuine development, as retrospective control was more adequate in the longitudinal than in the cross‐sectional sample. This finding is relevant because it suggests that metacognitive control, at least retrospective control, can be improved through repeated practice including retrospective control opportunities. Thus, retrospective control appears to be potentially trainable. Teachers should be encouraged to create test situations where children have the possibility to withdraw responses or correct responses with a differently colored pen. Then, not only performance could be graded, but additionally, adequate control decisions could be rewarded. The question remains when the acquired experience is sufficient to transfer it to different tasks and even more important to daily routines. Future research might target training of metacognitive control in elementary school children by considering different measures of control (prospective and retrospective).

Our findings suggest that metacognitive control is not a unitary skill. Rather, control skills appear to fluctuate and are highly sensitive to motivational aspects and task demands. Nevertheless, stability over time was moderate and did not differ significantly between prospective and retrospective control. Thus, under identical conditions as in this study, it is possible to predict accuracy of metacognitive control, at least to some extent. This indicates that poor metacognitive control skills can be expected to remain rather poor. Individuals falling behind in their metacognitive development might thus have difficulties catching up without intervention or specific, individualized instructions and feedback. This is another theoretically, but also practically important finding, owed uniquely to the longitudinal design of the present approach.

Although there was some improvement over time for control behavior, especially prospective control remained suboptimal. Overall, 42% of prospective control decisions in second graders and 41% in fourth graders were inadequate (misses and false alarms) at the second measurement point. For retrospective control, in contrast, inadequate control decisions were less frequent, but 31% were still inadequate in second graders, and 28% in fourth graders at T_2_, pointing to persisting metacognitive difficulties.

### Monitoring‐Based Control

As expected and based on the few prior studies, monitoring‐based control was superior in older compared to younger children both when looking at prospective and retrospective monitoring‐based control. Consistent with our expectations, prospective and retrospective monitoring‐based control improved in both age groups. Looking at the effect sizes reveals first evidence that the improvements over 1 year were more pronounced for retrospective than prospective monitoring‐based control, with the within‐subject correlations between CJs and retrospective control at T_2_ being relatively high (second graders: 0.74; fourth graders: 0.80). These results suggest that retrospective monitoring‐based control seems to be somewhat easier for elementary school children than prospective monitoring‐based control. The motivational effects on children’s restudy selection, discussed earlier, are likely to apply here, too. It is further conceivable that some children selected items with high JOLs for restudy instead of items with low JOLs because, as described earlier, this could be considered an effective strategy as well. However, while the comparison with the cross‐sectional sample in Study 2 revealed that retrospective monitoring‐based control was better in the longitudinal sample than in the cross‐sectional sample, there was no difference between the two samples regarding prospective monitoring‐based control. Thus, the observed improvements in prospective monitoring‐based control in second and fourth graders seem to reflect naturally occurring development. In contrast, the observed improvements in retrospective monitoring‐based control appear mainly due to practice and task familiarity. As was the case for retrospective control, children benefited from a repeated presentation and learned from their experience, with improvements being stronger for younger than for older children. In fact, second graders caught up with fourth graders at T_2,_ showing that the practice effect was stronger for second graders than for fourth graders. These findings are unique and suggest a critical period in elementary school during which monitoring‐based control may be especially vulnerable to experiences; this is highly relevant when tailoring interventions or when educating teachers.

### Persisting Metacognitive Difficulties

In this study, elementary school children showed suboptimal control, especially with regards to prospective control. The question remains unanswered what specifically makes the control of learning so difficult. With the explorative analyses regarding persisting metacognitive difficulties, we tried to find first answers to this question. Although there were indications for relatively adequate monitoring skills, a more detailed look revealed that monitoring was far from optimal.

First, monitoring judgments were more strongly associated with control than with actual recognition. Children seemed to use the middle category of the scale as a cut‐off for their control decisions and this cut‐off—unfortunately—appeared to be rather independent of actual performance. Moreover, children’s monitoring skills seemed limited as they only substantially discriminated in their monitoring judgments (JOLs and CJs) between correct and incorrect performance when *not* restudying an item or when *maintaining* a response. There are several speculative interpretations for these findings. Performance was overall higher for items that children decided not to restudy compared to items they restudied (36.6% vs. 26.7%), and for responses children maintained compared to those they withdrew (47.1% vs. 23.7%). Possibly, it is easier for children to monitor what they (believe to) know compared to when they are unsure (Roebers, 2017). Thus, the ability to monitor in a *relative* sense might pose specific problems (i.e., “Am I *more sure* for this item than for the previous one?”). For optimizing performance, it is more crucial to being able to evaluate uncertainty compared to certainty and this was found to be especially challenging for our participants (Finn & Metcalfe, 2014; Flavell, 2000). For teachers, it may thus be best to start with fostering monitoring and control skills on easy tasks that contain few difficult items. Children can then monitor and control adequately on the easy items and make contrasting experiences on the few difficult ones. This way, they make positive but also differentiated metacognitive experiences, which might enhance their motivation to engage in monitoring and increase their uncertainty monitoring skills in the long‐run. A second explanation for the inaccurate monitoring and control decision may lie in lucky guesses. That is, children may have been uncertain about the correct response and therefore indicated to restudy the item or withdrew the response not knowing or anticipating that they might have had a lucky guess.

Third, participants still showed signs of overconfidence in their monitoring, that is, their monitoring judgments were still overly optimistic when recognition was incorrect in an *absolute* sense. That is, mean JOLs for no restudy of an incorrect answer were around the middle category of the scale, 4.5 on the 7‐point scale, and mean CJs for an incorrect answer maintained were somewhat higher, 5 on the 7‐point scale. It appears that this overconfidence was a main reason for inadequate control. In other words, inadequate (overoptimistic) monitoring (prospective and retrospective) is a persisting metacognitive difficulty, both in children and adults (Dunlosky & Rawson, 2012). Children in their early elementary school years are able to make reasonable control decisions based on their monitoring, but as monitoring remains to be relatively inaccurate, control will also remain error‐prone. Thus, monitoring is the key aspect for the development of efficient metacognitive control.

Taken together, second and fourth graders were relatively well able to retrospectively monitor their learning, whereas to prospectively monitor their learning in a paired‐associate memory task with unfamiliar material posed greater difficulties. Prospective and retrospective monitoring seem to follow different developmental timetables, suggesting that different monitoring processes are involved. Children reliably based their metacognitive control on their monitoring judgments. However, a close inspection of the data revealed frequent incorrect control, which was found to be mainly a consequence of inadequate and overoptimistic monitoring. This study thus documents a possible starting point for improving self‐regulated learning. Focusing on retrospective metacognitive monitoring with the aim to optimize subsequent control may be an efficient way to improve self‐regulated learning, independent of age.
